# Cervicofacial cellulitis on vascular malformation: About two case reports

**DOI:** 10.1016/j.amsu.2021.102526

**Published:** 2021-06-30

**Authors:** Ulrich Opoko, Iro Salissou, Rachid Aloua, Mohamed Raiteb, Faiçal Slimani

**Affiliations:** a Faculty of Medicine and Pharmacy, Hassan II University of Casablanca, B.P, 5696, Casablanca, Morocco; b Department of Stomatology and Maxillofacial Surgery, Hospital 20 Aout, CHU Ibn Rochd, B.P, 2698, Casablanca, Morocco

**Keywords:** Cervicofacial cellulitis, Vascular malformation, Management

## Abstract

Cervicofacial cellulitis is a brutal and dreadful disease that poses a serious problem in terms of therapeutic management. Most often, these cellulitides occur either in a fragile environment or after inappropriate initial management. We report two (02) observations of cervicofacial cellulitis in patients with vascular malformation of the face. In both cases, the patients were previously diagnosed with a facial vascular malformation and presented with secondary cervicofacial cellulitis homolateral to the vascular malformation. The diagnosis of cellulitis was made by clinical and biological elements and confirmed by imaging (CT). This comorbidity remains a rare entity, and almost not reported in the literature; their management remains however complex and must be integrated with multidisciplinary consultations.

## Highlights

• Cervicofacial cellulitis is one of the most frequent infectious emergencies in maxillofacial surgery.
• They occur in fragile conditions most of the time.
• The comorbidity of cellulitis and vascular malformation remains a rare entity.
• Their management remains however complex and must be integrated with multidisciplinary consultations.

## 1 Introduction

Cervicofacial cellulitis is one of the most frequent infectious emergencies in Maxillofacial Surgery, but also one of the most serious and life-threatening. Most often, these cellulitides occur either in a fragile environment or after an inappropriate initial management [1]. We report here two cases of cervico-facial cellulitis occurring in a particular situation of vascular malformation of the face. Vascular malformations include lesions where there are abnormalities in vascular morphogenesis; the vessels are dysplastic without true cell proliferation [2]. The aim of this work is to show whether there is a correlation between the occurrence of cervicofacial cellulitis and vascular malformation.

## 2 Case reports

This article has been reported in line with the PROCESS criteria [3].

### 2.1 Case 1

**Fig. 1:**
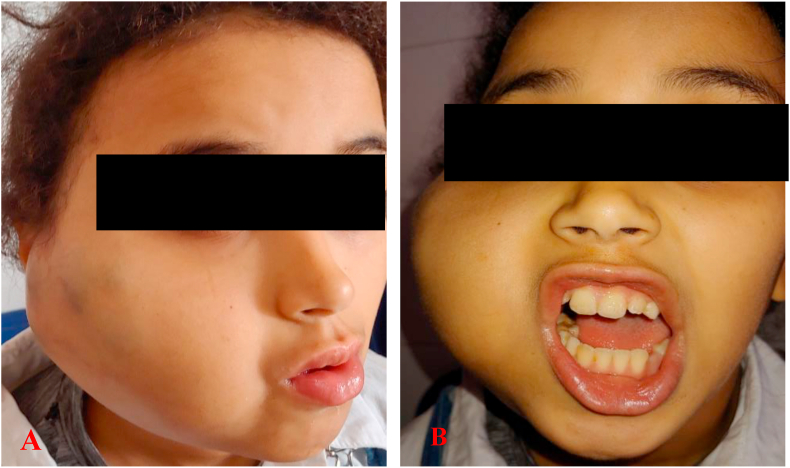
Picture showing the right jugal swelling with the bluish appearance opposite. B. Picture showing the trismus.

**Fig. 2:**
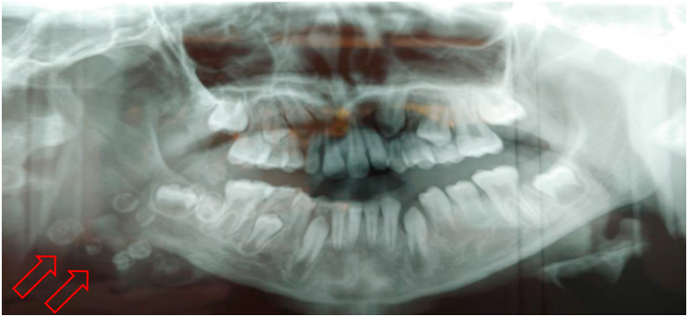
X-ray showing radiopaque images (phleboliths) in the right mandible.

**Fig. 3:**
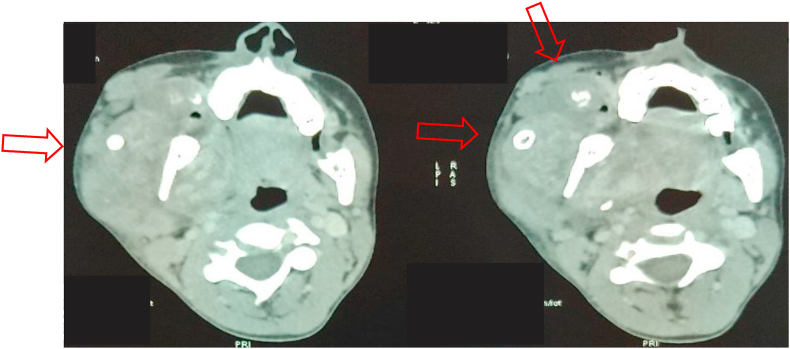
CT scan showing vascular malformation with macro calcifications.

A 10 year old girl, with no particular pathological history, followed since birth for a right jugal venous malformation, for which she had benefited three (03) times from sclerotherapy sessions, with a good evolution. One week before her admission, she presented a dental pain, treated only with analgesics, then appeared a right jugal swelling, becoming painful, thus motivating the consultation. The clinical examination revealed a right jugal swelling with a bluish aspect, soft, warm, painful to palpation (Fig. 1A). There was also a trismus (Fig. 1B), dental caries of the right mandibular second premolar, a fever of 38.5 °C, and a right sub-angulo-mandibular adenopathy. Biological examinations revealed an infectious syndrome (hyperleukocytosis at 12650/mm3 with granulocytic predominance, *C*-reactive protein at 46 ng/ml). The panoramic radiograph revealed multiple rounded, heterogeneous opacities of variable size, attached to calcifications located in the right angular area (Fig. 2). The facial CT scan confirmed the diagnosis of a venous type vascular malformation with a solid cystic process of the right hemiface with macro calcifications and inflammatory signs of temporal myositis (Fig. 3). Written informed consent was obtained from the parents of the minor girl who was treated with a bi-antibiotic therapy: amoxicillin + clavulanic acid 1 g x 2/day, metronidazole 500 mg x 2/day parenterally, and paracetamol 500 mg in case of pain or fever. The evolution was marked at D-2 by regression of the swelling and disappearance of the fever; at D-4 by an endobuccal fistulisation and pus. Discharge was made at D-7 with an oral relay and a letter to the dental faculty for dental care of the causal tooth. The total duration of treatment was 14 days.

### 2.2 Case 2

**Fig. 4:**
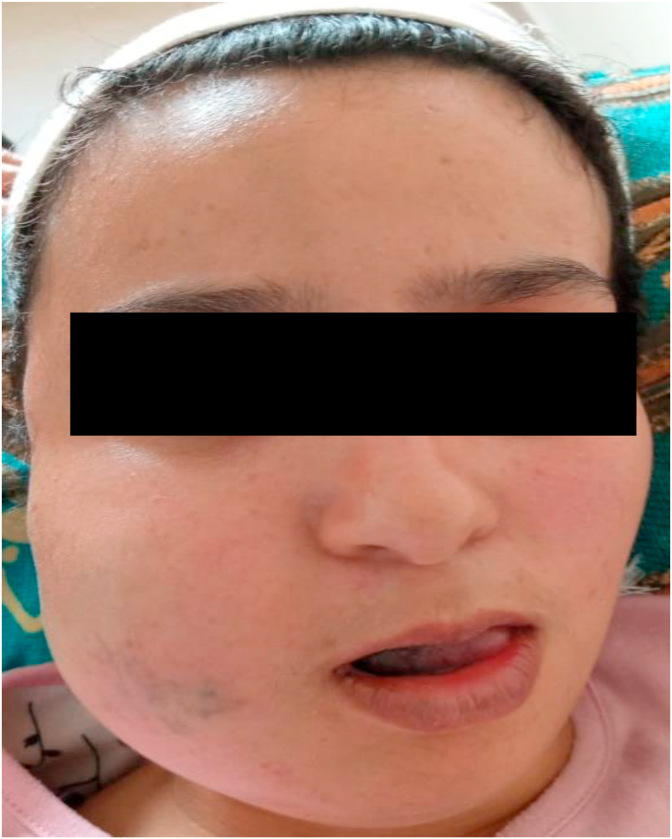
Picture showing the right jugal swelling and trismus.

A 22 year old female patient, followed for venous vascular malformation of the right cheek since childhood, on beta-blockers and who presented 5 days before her admission, a dental pain having been self-medicated with non-steroidal anti-inflammatory drugs (NSAIDs), responsible for a rapid increase of the jugal swelling, with some bluish spots, hot, painful, poorly limited, with trismus (Fig. 4) and fever at 39.2 °C. Endobuccal examination revealed dental caries on 48, with a bluish appearance of the right half tongue. Biological examinations revealed an infectious syndrome (hyperleukocytosis at 13400/mm3 with granulocytic predominance, a *C*-reactive protein at 76 ng/ml).

**Fig. 5:**
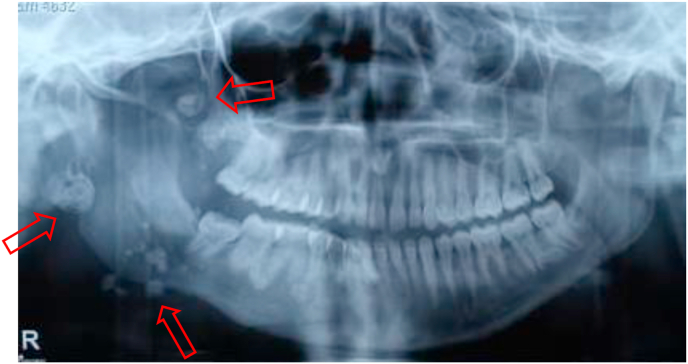
X-ray showing pheboliths in the right mandible with caries in the 48.

**Fig. 6:**
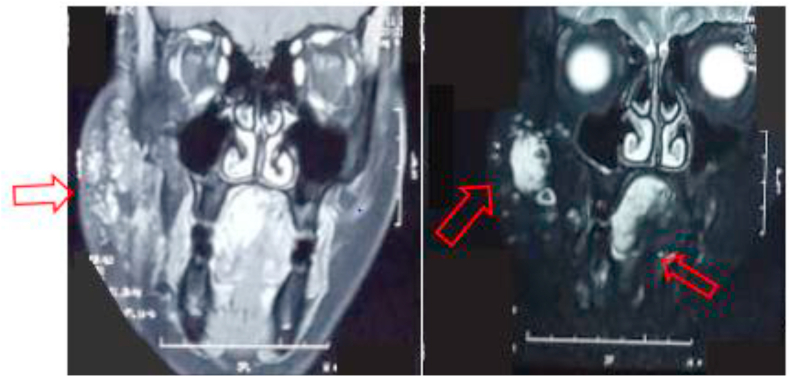
CT scan showing venous vascular malformation at right jugal and lingual level with some phleboliths.

The panoramic radiograph showed calcifications in the right angular region, with confirmation of caries in the 48 (Fig. 5). The CT scan of the face confirmed the diagnosis of cellulitis of the right jugal soft tissue with extension to the deep spaces of the face without a clearly individualised collection of the facial venous malformation (Fig. 6). The patient was also treated with a bi-antibiotic therapy: amoxicillin + clavulanic acid 1 g x 3/day, metronidazole 500 mg x 3/day parenterally, and paracetamol 1000 mg in case of pain or fever. The evolution was marked at D-3 of treatment by the regression of the swelling, and the disappearance of the fever; at D-5 one noted a fistulisation in endobuccal and from the pus in the vestibular level opposite the 48. Extraction of the decayed tooth was done at D-7 after improvement of the mouth opening and the exit was done at D-8 with a peros relay. The total duration of treatment was 10 days.

## 3 Discussion

Cervicofacial cellulitis is a serious and life-threatening condition [4,5]. They pose a real problem for therapeutic management [1]. The association of cellulitis and vascular malformation is a very rare entity and hardly described in the literature. In our series, we report two cases of subjects who were carriers of a vascular malformation of the face and who presented with cervico-facial cellulitis on the same side of the vascular malformation.

Vascular malformations are part of a large family of vascular anomalies; a classification of vascular anomalies proposed by the International Society for the Study of Vascular Anomalies (ISSVA), based on the work of Mulliken and Glowaski, has provided a clear and schematic distinction between vascular malformations and vascular tumours [6,7]. These malformations are classified according to histological and haemodynamic characteristics (slow or fast flow). A distinction is made between slow-flow malformations, which, depending on the predominantly altered vessels, are capillary, venous or lymphatic, and fast-flow malformations, which are arteriovenous malformations [2]. Venous malformations have a slow flow; they consist of dysplastic veins whose walls are deficient in smooth muscle cells [8]. They are the most frequent vascular malformations, accounting for 2/3 of cases [9].

In our case series, these were patients who had been diagnosed with vascular malformations for several years and for which they were being followed up; in both cases, it was a venous vascular malformation. The first had benefited from three (03) embolization sessions (sclerotherapy) with a favourable and stable evolution while the second was treated with beta-blockers with regular monitoring.

The diagnosis of cervicofacial cellulitis is clinical, based on the conjunction of a severe infectious condition and physical cervicofacial signs. Cervicofacial swelling is almost always inflammatory and painful. On its own, it is highly suggestive of cellulitis. The association with trismus and odynophagia is usual [10,11]. These clinical elements were found in our cases, with the exception of odynophagia.

Biologically, there is a moderate hyperleukocytosis with a predominance of neutrophils [12]. Our results concur with this finding. The CT scan allowed us to confirm the diagnosis of cervico-facial cellulitis on the venous type vascular malformation and to see its loco-regional extension.

The portal of entry is dental or peridental in more than 80% of cases [[13], [14], [15]], as was the case in our two cases where the dental aetiology of the cellulitis was incriminated. This high incidence is due to poor oral hygiene [5,10,13]. The germs involved are variable; they are most often saprophytic germs of the oral cavity. The predominance of anaerobic germs is unanimously accepted by authors [16,17].

The treatment is medical and surgical associated with an adapted resuscitation [1]. Given the poly-microbial nature of this infection, it is important to start broad-spectrum probabilistic antibiotic therapy as soon as possible, while awaiting the results of bacteriological samples and the antibiogram [18]. The duration varies according to the degree of severity and evolution. Surgical drainage is an essential part of the treatment and must allow all cellulitis zones to be flattened by a wide approach [1].

In our series, the patients only benefited from medical treatment based on bi-antibiotic therapy with amoxicillin + clavulanic acid and metronidazole, with a favourable evolution marked by an endobuccal fistulisation with the release of pus, a defervescence of the fever, a regression of the swelling and the trismus. Bacteriological analysis of the pus, for adjustment of antibiotic therapy, could not be performed, due to the difficulty of endobuccal sampling, as the fistulisation in these two cases was done endobuccally. Stomatological treatment is usually performed after cooling of the infectious process [11], as was the case in our series. Surgical drainage was not performed, due to the good response of our patients to medical treatment, but it should also be noted the complexity of performing a surgical procedure in these particular sites with a vascular malformation homolateral to the cellulitis.

In the literature, we have not found a correlation between the occurrence of cellulitis and vascular malformation, however, certain favouring factors have been described in the occurrence of cellulitis, among which are non-steroidal anti-inflammatory drugs (NSAIDs), which are often considered as the main favouring factors of cervico-facial cellulitis [1,11,[18], [19], [20]]; factors associated with circulatory problems, namely the presence of oedema (particularly lymphoedema), venous insufficiency and a history of cellulitis or surgery affecting lymphatic drainage; and other factors such as obesity, smoking [21]. However, the vascular malformation present in both patients was a venous type vascular malformation, which is characteristically slow flowing [2], due to the vascular dysplasia that they originally caused the circulatory disorder, and therefore venous insufficiency. These types of venous malformations can be painful due to the tension of the malformation or due to the presence of calcifications, or phleboliths [2], as found in our two cases, which can lead to confusion if they were pains related to cellulitis alone, or to those related to calcifications or to both. At the facial level, these malformations can infiltrate the muscles and the different spaces (pterygomaxillary fossa, orbit …) [2,25]. In the first case, there was tissue infiltration of the jugal area, the right infra-temporal fossa, the homolateral parapharyngeal, prestylial and retrostylial spaces, the deep laterocervical region and the floor of the mouth, whereas in the second case, the infiltration was of the soft tissues of the jugal area, the right infra-temporal area and the right hemi-lipid. There is no correlation between the size of the malformation on the surface and its extension in depth [2].

The treatment of venous malformations depends on their location and volume. Percutaneous sclerosis can be used alone or in combination with surgery [2]. This treatment consists of an injection of various sclerosing products (Ethibloc®, OK-432 or picibanil killed bacteria) [22,23], in order to reduce the volume of the vascular malformation. Surgery is required for venous malformations with functional repercussions such as endobuccal and labial locations [2].

The prognosis of cervicofacial cellulitis is essentially related to the terrain; the precocity and effectiveness of the initial treatment. The percentage of deaths in the literature varies between 7 and 50% [12,24]. In our series, we noted no deaths, the patients benefited from a regular follow-up after their discharge: at D-14, 1 month, 3 months and 6 months, with a good evolution and without recurrence of the cellulitis, and stabilization of their vascular malformation.

Concerning the vascular malformations that the two patients present, and their extensions for the two cases, in the deep spaces of the face, a follow-up is programmed at the consultation every 6 months, marked for a simple monitoring in order to appreciate the evolution of the jugal swelling related to the vascular malformation, and a surgery will be envisaged only in the event of important functional discomfort, preceded as a preliminary by an embolization 24 h before the surgery.

## 4 Conclusion

The co-morbidity of vascular malformation and cervicofacial cellulitis is a very rare entity and hardly described in the literature, indeed it is difficult to establish a correlation of cause and effect of the occurrence of cervicofacial cellulitis in these patients with vascular malformations. However, the type of vascular malformation reported in our series was venous, known to be slow flowing; and in the literature among the risk factors described in the occurrence of cellulitis, we note circulatory problems, including venous insufficiency. The management of these anomalies remains complex and must be integrated into multidisciplinary consultations.

## Patients concent

Written informed consent was obtained from the parents of the minor girl and the second patient for the publication of this case report and accompanying images. A copy of the written consents is available upon request for review by the editor of this journal.

## Provenance and peer review

Not comissioned, externally peer reviewed.

## Please state any conflicts of interest

Authors of this article have no conflict or computing interest.

## Please state any sources of funding for your research

None.

## Ethical approval

Our study is exempted of ethical approval.

## Consent

Written informed consent was obtained from both patients for publication of this case report and accompanying images. A copy of the written consents is available for review by the Editor-in-Chief of this journal on request”.

## Author contribution

Ulrich OPOKO: study concept, data collection, writing the paper and making the revisions following the reviewer's instructions. Iro SALISSOU: revision following the reviewer's instructions. Rachid Aloua: writing the paper. Mohamed Raiteb: writing the paper. Faiçal SLIMANI: reviewing and validating the manuscript's credibility.

## Registration of research studies

1. Name of the registry:
2. Unique Identifying number or registration ID:
3. Hyperlink to your specific registration (must be publicly accessible and will be checked):

## Guarantor

Ulrich OPOKO.

## Declaration of competing interest

Authors of this article have no conflict or computing interest.
